# Extracellular proteolytic enzymes produced by human pathogenic *vibrio* species

**DOI:** 10.3389/fmicb.2013.00339

**Published:** 2013-11-18

**Authors:** Shin-ichi Miyoshi

**Affiliations:** Graduate School of Medicine, Dentistry and Pharmaceutical Sciences, Okayama UniversityOkayama, Japan

**Keywords:** *Vibrio*, vibriolysin, thermolysin, collagenase, serine protease

## Abstract

Bacteria in the genus *Vibrio* produce extracellular proteolytic enzymes to obtain nutrients via digestion of various protein substrates. However, the enzymes secreted by human pathogenic species have been documented to modulate the bacterial virulence. Several species including *Vibrio cholerae* and *V. vulnificus* are known to produce thermolysin-like metalloproteases termed vibriolysin. The vibriolysin from *V. vulnificus*, a causative agent of serious systemic infection, is a major toxic factor eliciting the secondary skin damage characterized by formation of the hemorrhagic brae. The vibriolysin from intestinal pathogens may play indirect roles in pathogenicity because it can activate protein toxins and hemagglutinin by the limited proteolysis and can affect the bacterial attachment to or detachment from the intestinal surface by degradation of the mucus layer. Two species causing wound infections, *V. alginolyticus* and *V. parahaemolyticus*, produce another metalloproteases so-called collagenases. Although the detailed pathological roles have not been studied, the collagenase is potent to accelerate the bacterial dissemination through digestion of the protein components of the extracellular matrix. Some species produce cymotrypsin-like serine proteases, which may also affect the bacterial virulence potential. The intestinal pathogens produce sufficient amounts of the metalloprotease at the small intestinal temperature; however, the metalloprotease production by extra-intestinal pathogens is much higher around the body surface temperature. On the other hand, the serine protease is expressed only in the absence of the metalloprotease.

## HUMAN PATHOGENIC *VIBRIO* SPECIES

Bacteria in the genus *Vibrio* are normal habitants of aquatic environments and play important roles in maintaining the aquatic ecosystem. Although more than 100 species are currently in this genus, at least, 11 species listed in **Table 1** are human pathogens (Janda et al., 1988; Chakraborty et al., 1997). Amongst, *Vibrio hollisae* is recommended to place into a novel genus *Grimontia* (Thompson et al., 2003). Human pathogenic species can be classified into two groups according to types of infectious diseases: the group causing gastrointestinal diseases and that causing extra-intestinal diseases. Representative species in the former group are *V. cholerae* and *V. parahaemolyticus*, whereas *V. vulnificus* is the most important species in the latter group. Two species, *V. damsela* and *V. vulnificus*, are also fish pathogens.

**Table 1:** Infectious diseases caused by *Vibrio* species.

Species	Intestinal diseases		Extra-intestinal diseases		
	Cholera	Diarrhea	Septicemia	Wound-infection	Others*
*V. alginolyticus*			±	++	±
*V. cholerae* O1/O139**	++				
*V. cholerae* non O1/ non O139		++	+	+	±
*V. cincinnatiensis*					±
*V. damsela*				+	
*V. fluvialis*		++			
*V. furnissii*		±			
*V. hollisae*		±			
*V. metschnikovii*		±			±
*V. mimicus*		++			±
*V. parahaemolyticus*		++	+	++	±
*V. vulnificus*		±	++	++	±

*++, Major disease; +, Minor disease; ±, Rare disease.*

* Include otitis media, cholesystitis, meningitis

Of human pathogenic vibrios, *V. cholerae* is the most extensively studied species because it is a causative agent of severe watery diarrheal disease, cholera. Cholera is characterized by uncontrolled purging of copious rice water stools leading to serious electrolyte depletion, dehydration, acidosis, shock, and, if left untreated, to death. Although *V. cholerae* is divided serologically into more than 200 groups, only two serogroups, O1 and O139, are etiologic agents of epidemic cholera. The non-O1/non-O139 serogroups are etiologic agents causing sporadic diarrheal cases and occasionally extra-intestinal infections, including skin, ear, sputum, urine, and cerebrospinal fluid infections. Cholera toxin (CT) produced by the O1 and O139 serogroups is a major toxic factor evoking severe watery diarrhea, whereas, hemolysin (Shinoda and Miyoshi, 2006), Zonula occludens toxin (Fasano et al., 1991), and accessory CT (Trucksis et al., 1993) have also been demonstrated to be additional enterotoxic factors.

*Vibrio parahaemolyticus* inhabits commonly coastal and estuary areas in the tropical and temperate regions, but this species has been recognized to cause gastroenteritis following consumption of seafood. The outstanding features of gastroenteritis are severe abdominal pain, diarrhea (frequently bloody stools), nausea, vomiting, mild fever, and headache. The mean incubation period is 6 to 12 h and diarrhea or soft stools persist for 4–7 days. Although the food poisoning caused by *V. parahaemolyticus* had been frequent in Japan, the number of outbreaks has been decreased drastically in the last 10 years. Production of the hemolysin designated as thermostable direct hemolysin is closely related to the bacterial pathogenicity (Shinoda and Miyoshi, 2006). *V. parahaemolyticus* also causes wound-infection through exposure of a new wound to contaminated seawater or estuarine water, whereas this type of diseases is independent of production of the hemolysin.

The first clinical isolation of *V. vulnificus* was from a human leg ulcer (Roland, 1970). However, because of similar bacteriological characteristics of the bacterium isolated, this case was reported as an extra-intestinal infectious disease by *V. parahaemolyticus* (Hollis et al., 1976). *V. vulnificus* causes two types of infections, the primary septicemia and the wound-infection, to human (Blake et al., 1980; Klontz et al., 1988). The primary septicemia is associated with consumption of contaminated raw seafood, especially shellfish such as oysters. However, this type is a typical opportunistic infection. Namely, most patients have an underlying disease(s) of liver dysfunction, alcoholic cirrhosis or hemochromatosis, which leads to increase in the plasma ferric ion level and to decrease in the activity of the innate immunity (Strom and Paranjpye, 2000; Inoue et al., 2008). In two-thirds of the patients, the edematous and/or hemorrhagic secondary skin lesions appear on the extremities and the trunk (Blake et al., 1980; Inoue et al., 2008). The wound-infection is characterized by the development of edema, erythema or necrosis around a new wound exposed to seawater contaminated with *V. vulnificus* (Blake et al., 1980; Klontz et al., 1988). This type of infection can occur in healthy persons, as well as in the immuno-compromised hosts, and may occasionally progress to septicemia.

## BACTERIAL EXTRACELLULAR PROTEOLYTIC ENZYMES

Human pathogenic vibrios produce various extracellular factors including enterotoxin, hemolysin, cytotoxin, protease, collagenase, phospholipase, siderophore, and hemagglutinin (Janda et al., 1988). Of these factors, enterotoxin, hemolysin, and cytotoxin are directly related to the clinical symptoms; however, siderophore and hemagglutinin may play roles in the establishment of the infection.

Proteolytic enzymes hydrolyzing a peptide bond in proteins and peptides are essential for the homeostatic control in both eukaryotes and prokaryotes, and thus, the bacterial enzymes also have various physiological roles in the life cycle of the microorganisms. However, the enzymes produced by pathogenic species occasionally act as toxic factors to the infected host (Hase and Finkelstein, 1993; Harrington, 1996). Proteolytic enzymes are classified into several groups, such as aspartic, cysteine, and serine protease, and metalloprotease; however, many of the bacterial toxic proteases are in the metalloprotease group, which often contains a zinc (II) ion in the catalytic center (Hase and Finkelstein, 1993; Hooper, 1994). As shown in **Table 2**, human pathogenic *Vibrio* species also produce and secrete proteolytic enzymes, and several enzymes have been extensively characterized as direct toxic factors causing skin damage or indirect virulence factors processing other protein toxins. The enzymes produced by vibrios are in two metalloprotease groups (vibriolysin and collagenases) or one serine protease group (chymotrypsin-like proteases).

**Table 2:** Extracellular proteolytic enzymes produced by pathogenic *Vibrio* species.

Species	Metalloprotease		Serine protease
	Vibriolysin	Collagenase	Chymotrypsin-like protease
*V. alginolyticus*		+	+
*V. cholerae*	+		
*V. fluvialis*	+		
*V. metschnikovii*			+
*V. mimicus*	+		
*V. parahaemolyticus*		+	+
*V. vulnificus*	+		+

The progress in the molecular biology has provided much information on the DNA-derived amino acid sequences of metalloproteases and has shown the presence of the consensus sequence HEXXH as the zinc-binding motif. This motif was also found in some bacterial protein toxins including clostridial neurotoxins, *Bacteroides fragilis* enterotoxin, and *Bacillus anthracis* lethal factor. Indeed, these bacterial toxins were verified to show the remarkably specific proteolytic action toward a target host protein (Miyoshi and Shinoda, 2000). For instance, clostridial neurotoxins can cleave the restricted protein components of the neuroexocytosis machinery, which leads to the blockade of neurotransmitter release and consequent muscle paralysis (Schiavo et al., 1993). In addition, a novel cytotoxin that consists of one A subunit and five B subunits was isolated from some enterohemorrhagic *Escherichia coli* strains, and the A subunit was indicated to be a subtilase-like serine protease (Paton and Paton, 2010). The RTX (repeated-in-toxin) toxins are large multifunctional cytotoxins and are possible to modulate the virulence of a number of gram-negative bacterial pathogens including *V. cholerae* and *V. vulnificus* (Satchell, 2007; Prochazkova et al., 2009; Roig et al., 2011). In *V. cholerae* RTX toxin, the cysteine protease domain was reported to mediate autoprocessing of the toxin (Sheahan et al., 2007; Shen et al., 2009).

## VIBRIOLYSIN

### BIOCHEMICAL PROPERTIES

Zinc-containing metalloproteaes consist of four superfamilies based on the amino acid residues in the zinc-binding site, and the zincins superfamily is characterized by the possessing of the HEXXH motif (Hooper, 1994). The thermolysin family, in which prototype enzyme is thermolysin from *Bacillus thermoproteolyticus*, is a one of major members of this superfamily. The enzymes in this family are commonly characterized by the presence of Glu at the 25th position from the first His of the above motif. *V. proteolyticus* is a marine microorganism that was first isolated from the intestine of a small, wood-boring isopod crustacean (Merkel et al., 1964). Griffin and Prescott (1970) purified a highly active metalloprotease from the bacterial culture supernatant. Durham (1989) first disclosed the designation of this enzyme as vibriolysin in the patent literature. Thereafter, highly homologous metalloproteases have been isolated from other *Vibrio* species including human pathogens *V. cholerae*, *V. fluvialis*, *V. mimicus*, and *V. vulnificus*. Therefore, the name of vibriolysin is currently applicable to all of these proteolytic enzymes (Miyoshi et al., 2012a).

Vibriolysin hydrolyzes specifically the peptide bond at the amino group side of the P1′ amino acid residue, which is usually a hydrophobic amino acid reside (e.g., Phe, Tyr, or Leu; Narukawa et al., 1993). Synthetic oligopeptides, such as carbobenzoxy (Z)-Gly-Phe-NH_2_ and Z-Gly-Leu-NH_2_, are thus commonly used as the suitable substrate. On the other hand, phosphoramidon [*N*-(α-rhamnopyranosyloxy-hydroxyphosphanyl)Leu-Trp] and zincov [2-(*N*-hydroxycarboxamido)-4-methylpentanoyl-Ala-Gly amide] are well-known competitive inhibitors. In addition, phenylazobenzyloxycarbonyl-Pro-Leu-Gly-Pro-Arg, the substrate developed for bacterial collagenases, is significantly hydrolyzed by the enzyme (Miyoshi et al., 1987b). Vibriolysin is also highly active on a wide variety of protein substrates. Namely, the enzyme exhibits significant proteolysis of casein, albumin, hemoglobin, type I and IV collagen, gelatin, elastin, fibrin, and fibrinogen (Miyoshi et al., 1995; Miyoshi and Shinoda, 2000).

**FIGURE 1:**
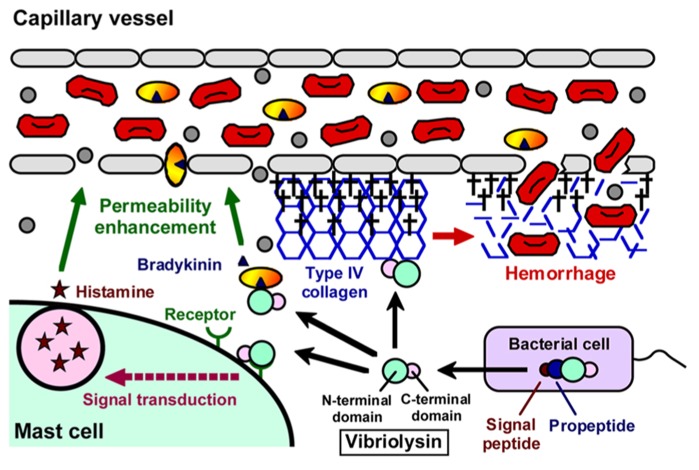
**Domain structure of vibriolysin, and direct toxic roles of vibriolysin in the formation of the hemorrhagic brae**.

Like thermolysin, vibriolysin is synthesized as an inactive precursor, and maturation of the precursor is achieved by several processing stages (Chang et al., 2007). In the case of the enzyme from *V. vulnificus* (**Figure 1**), it is initially synthesized as the preproenzyme (609 aa, 65,964 Da) with a typical signal peptide (Cheng et al., 1996). The signal peptide is cleaved during its passage through the inner membrane in the signal peptidase-dependent manner. In the periplasm, the propeptide that may function as an intramolecular chaperone mediating maturation of the enzyme and/or a specific inhibitor to protect autodigestion of the enzyme (Chang et al., 2007) is then cleaved by an autocatalytic mechanism, and finally, the mature vibriolysin (413 aa, 44,648 Da) is generated. The vibriolysin maturated consists of two functional domains: the N-terminal domain (314 aa, 34,049 Da) mediating the catalytic action, and the C-terminal domain (99 aa, 10,656 Da) essential for efficient attachment to protein substrates (Yun et al., 2012). The N-terminal domain is easily obtained by autocatalytic limited-digestion of the C-terminal domain (Miyoshi et al., 1997). The N-terminal domain alone possesses sufficient proteolytic activity toward oligopeptides or soluble proteins, while it shows markedly reduced activity toward insoluble proteins such as type I collagen and elastin.

The N-terminal domain of vibriolysin is significantly related to other enzymes in the thermolysin family; however, its C-terminal domain may be unique. The feature of the proteolytic action, which is mediated by the N-terminal domain, is considerably similar to other enzymes. However, neither suitable specific peptide substrate nor competitive inhibitor for vibriolysin has been developed. On the other hand, the hemagglutinating action on rabbit erythrocytes, which is due to association of both N-terminal and C-terminal domains with the erythrocyte membrane, is a distinctive feature of vibriolysin so far as reported (Alam et al., 1995).

### PATHOLOGICAL ROLES

**FIGURE 2:**
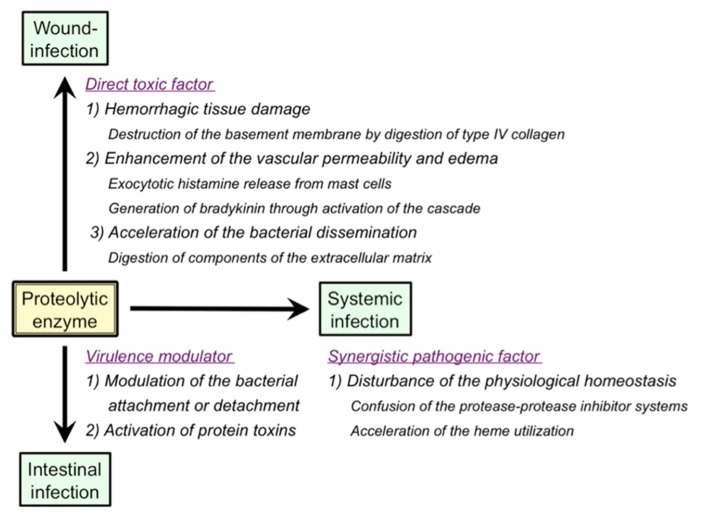
**Pathological roles of extracellular proteolytic enzymes in infectious diseases caused by *Vibrio* species**.

As summarized in **Figure 2**, a variety of pathological roles of vibriolysin have been documented (Shinoda and Miyoshi, 2011). In the local infections such as the wound-infection, vibriolysin is thought to be a direct toxic factor that causes hemorrhagic tissue damage through digestion of the basement membrane around vascular endothelial cells, and that forms edematous lesions through generation of inflammatory mediators (**Figure 1**). The enzyme from *V. vulnificus* can enhance the vascular permeability when injected into the mammalian dorsal skin. In rat skin, this reaction is due to the release of histamine from mast cells, because the vascular enhancement was abolished by simultaneous injection of diphenhydramine, an anti-histaminic agent (Miyoshi et al., 1987c). In guinea pig skin, however, the permeability enhancement is most likely due to activation of the factor XII-plasma kallikrein-kinin cascade (Miyoshi et al., 1987a). Namely, the skin reaction was not blocked by diphenhydramine, but it was modulated by *in situ* administration of the specific inhibitors affecting the cascade activation. Bradykinin, a well-known *in vivo* mediator of inflammation, is finally generated through activation of the cascade. Further studies to clarify the activation mechanism of the human cascade have been carried out, and the results demonstrated that vibriolysin could generate the active forms via limited proteolysis of the inactive zymogens (Miyoshi et al., 2004). Plasma prekallikrein was converted to the active kallikrein, which can liberate bradykinin from kininogen, by cleavage of the Arg^371^-Ile^372^ bond. On the other hand, factor XII was activated by hydrolysis of the Arg^353^-Val^354^ or Gly^357^-Leu^358^ bond, and activated factor XII could convert plasma prekallikrein to kallikrein. Vibriolysin also induces the hemorrhagic reaction in the mammalian dorsal skin. The enzyme from *V. vulnificus* showed the greatest hemorrhagic activity compared with some bacterial metalloproteases, thermolysin from *B. thermoproteolyticus*, serralysin from *Serratia* species, and collagenase from *Clostridium histolyticum* (Miyoshi et al., 1998). The levels of the *in vivo* hemorrhagic activities of these proteases were correlated with those of the *in vitro* proteolytic activities toward the reconstituted basement membrane gel. Of two major basement components, laminin and type IV collagen, only the latter was easily digested by vibriolysin. This indicates that type IV collagen forming the framework of the basement membrane is the target protein. Therefore, specific degradation of type IV collagen causes destruction of the basement membrane, breakdown of capillary vessels, and finally the leakage of blood components including erythrocytes.

In the systemic infections including septicemia, vibriolysin may act as a synergistic pathogenic factor through disordered proteolysis of various plasma proteins, which in turn disturbs the physiological homeostasis, and eventually elicits an immuno-compromised state in the infected host. For instances, vibriolysin has been documented to facilitate the bacterial infection by disturbance of the plasma protease-protease inhibitor systems (Miyoshi et al., 1995), and to interfere with the blood homeostasis through prothrombin activation and fibrinolysis (Chang et al., 2005; Kwon et al., 2007). Acceleration of the heme utilization was also reported as the pathogenic role of vibriolysin (Nishina et al., 1992). Ferric/ferrous ion is essential for *in vivo* growth of pathogenic microorganisms. However, the concentration of free ferric/ferrous ion in human body is very low (10^-^^15^ to 10^-^^18^ M) because of the presence of heme-proteins including hemoglobin and iron-binding proteins. Therefore, pathogenic microorganisms invaded into human body must operate the systems to acquire ferric/ferrous ion. Heme, a complex of porphyrin with ferric/ferrous ion and a prosthetic group in hemoglobin or other heme-proteins, is often used as an iron source by human pathogenic bacterial species (Stojiljkovic and Perkins-Balding, 2002; Tong and Guo, 2009). Nishina et al. (1992) documented that the vibriolysin-deficient mutants of *V. vulnificus* could not grow in the iron-restricted broth because of the inability to utilize hemoglobin as an iron source, but the bacterial growth was apparently restored by the addition of the purified vibriolysin. On the other hand, hemoglobin or ferric ion is known to be required for efficient transcription of the gene encoding the vibriolysin (Kawase et al., 2004; Sun et al., 2006). Incidentally, it should be noted that human plasma contains a broad-range protease inhibitor α_2_-macroglobulin (α_2_ M) as a primary inhibitor for exogenous proteolytic enzymes including bacterial enzymes. Vibriolysin was also inactivated with α_2_ M at a molar ratio of 1:1 by means of physical entrapment (Miyoshi and Shinoda, 1989). Therefore, the pathological actions of vibriolysin documented herein are possible to support the systemic infection but may be highly restricted *in vivo*.

Vibriolysin from enteric pathogens may increase the bacterial attachment to the intestinal surface through digestion of the mucosal substances or the bacterial outer membrane proteins (Alam et al., 2006). However, the inverse effect of the enzyme was also known. The vibriolysin from *V. cholerae* O1 was reported to accelerate the bacterial detachment from cultured cells by digestion of *V. cholerae* adhesins (Finkelstein et al., 1992). Vibriolysin also modulate the enterotoxicity of the bacterial toxins by limited proteolysis. For instance, the enzyme from *V. cholerae* O1 can activate CT through nicking of the A subunit of CT (Booth et al., 1984). Vibriolysin also converts the precursor of the enterotoxic hemolysin produced by *V. cholerae* to the mature toxin through removal of the 15 kDa N-terminal propeptide (Nagamune et al., 1996). Although the results shown above suggest indirect pathogenic roles of vibriolysin, the possibility of the direct roles has also been reported. Ghosh et al. (2006) purified vibriolysin from a CT gene-negative strain of *V. cholerae* non-O1/non-O139 and measured the enterotoxic activity. The purified enzyme caused accumulation of the hemorrhagic fluid in the rabbit ileal loop assay and increase in the intestinal short-circuit current in the Using chamber assay. Additionally, through the analysis with several mutants genetically constructed, Silva et al. (2006) showed that vibriolysin was necessary for full expression of enterotoxicity of *V. cholerae* O1.

## COLLAGENASES

Yu and Lee (1999) and Kim et al. (2002) carried out cloning of the *V. parahaemolyticus* gene encoding a collagenase, which was designated as PrtV/PrtVp (562 aa, 63,156 Da) and VppC (814 aa, 89,833 Da), respectively. These enzymes were revealed to be metalloproteases in the zincins superfamily having the consensus zinc-binding HEXXH motif, but neither of the collagenases was in the thermolysin family. Miyoshi et al. (2008) showed that, only when *V. parahaemolyticus* was cultivated at 26°C, the *vppC* gene was sufficiently expressed, and VppC was secreted from the bacterial cell after removal of the N-terminal 72 amino acid residues. In contrast, expression of the *prtV/prtVp* gene was negligible in the wild type strain. The gene was significantly expressed by disruption of the *vppC* gene; however, the product PrtV/PrtVp was not secreted into the cultivation broth (Miyoshi et al., 2008), suggesting PrtV/PrtVp is a cell-associated enzyme. VppC purified showed the steady activity to hydrolyze Z-Gly-Pro-Gly-Gly-Pro-Ala, the specific substrate for bacterial collagenases, and to digest gelatin. This indicates that VppC may contribute to the wound-infection by *V. parahaemolyticus* because putative digestion of the components of the extracellular matrix by VppC may accelerate the bacterial dissemination and may form cellulitic skin damage. *V. alginolyticus*, another species causing the wound-infection, is known to produce a VppC homolog (Takeuchi et al., 1992).

## CHYMOTRYPSIN-LIKE PROTEASES

In 2002, two research groups reported individually purification of a serine protease, which was termed protease A and VPP1 respectively, from the culture supernatant of *V. parahaemolyticus* (Ishihara et al., 2002; Lee et al., 2002). These proteases were identical and corresponded to the VPA0227 protein (677 aa, 71,038 Da) of strain RIMD 2210633 (GenBank accession number: BA000032). However, the amino acid sequencing of the purified enzyme indicated that the N-terminal 121 amino acid residues were removed during the maturating process. This serine protease designated herein as protease A/VPP1 showed the immunological cross-reactivity with serine proteases from *V. metschnikovii* and *V. alginolyticus* (Ishihara et al., 2002). Indeed, protease A/VPP1 revealed highly similarity of the amino acid sequence to the enzyme from *V. metschnikovii* (Kwon et al., 1995). Production of protease A/VPP1 was much higher at 25 than 37°C and was induced by the addition of gelatin to the cultivation broth. Miyoshi et al. (2008) reported that production of this serine protease was remarkably increased by disruption of the *vppC* gene, suggesting that protease A/VPP1 is a substitutive enzyme of VppC. The purified protease was found to be sensitive to chymostatin, the well-known competitive inhibitor for chymotrypsin, and to hydrolyze the specific peptidyl-4-methyl-coumaryl-7-amide (MCA) substrates for chymotripsin, such as Glutaryl-Ala-Ala-Phe-MCA and Succinyl-Ala-Ala-Pro-Phe-MCA. Therefore, it may be concluded that protease A/VPP1 is a chymotrypsin-like serine protease. The cytotoxicity against CHO, HeLa, or Vero cells and the mouse lethal toxicity of purified protease A/VPP1 was demonstrated by Lee et al. (2002). Our preliminary study showed the proteolytic activity of the purified enzyme toward extracellular matrix components, laminin and type I collagen. These results suggest that protease A/VPP1 also modulate the bacterial pathogenicity.

*Vibrio vulnificus* sometime causes severe hemorrhagic septicemia called vibriosis in eels of the culture farms (Tison et al., 1982; Biosca et al., 1991). A few strains isolated from diseases eels were recently found to have lost the 80 kb genomic region including the gene encoding vibriolysin, but instead of vibriolysin, these strains secrete a serine protease termed VvsA, which is an ortholog of protease A/VPP1 from *V. parahaemolyticus* (Miyoshi et al., 2012b). As protease A/VPP1, production of VvsA was extremely increased in the absence of the functional gene encoding vibriolysin (Wang et al., 2008). The *vvsA* gene constitutes an operon with a downstream gene *vvsB*, of which product VvsB may act as the chaperon supporting the maturation process of VvsA. The database analysis showed that several *Vbrio* species including *V. parahaemolyticus* have the homologus genes to *vvsAB*, indicating widely distribution of the chymotrypsin-like serine protease in *Vibrio* species.

## REGULATION OF PRODUCTION OF PROTEOLYTIC ENZYMES

*Vibrio* species are ubiquitous microorganisms in aquatic environments, but 11 species cause intestinal or extra-intestinal infections to humans. During the infection process, the bacterial cells must sense the change of environmental factors, such as temperature, pH, salinity, and osmolarity, and then, the bacterium must transmit the signals into the cells through the specific signal-transduction systems, which result in the change of expression of the genes. Especially, the genes encoding the toxic or virulence factors, which may be required for *in vivo* survival and growth, must be expressed at an appropriate place and time in a tightly regulated fashion (Heithoff et al., 1997; Lee et al., 1999). Amongst the environmental factors, temperature is thought to be the most important. The vibriolysin genes of the bacterium causing intestinal infections are expressed sufficiently at 37°C, while the genes of the bacterium causing extra-intestinal infections are expressed more effectively around the body surface temperature (Watanabe et al., 2004; Miyoshi et al., 2006). The gene expression is also often affected by the salinity. For instance, the expression level of the *vppC* gene in *V. parahaemolyticus* is higher at 3% NaCl than 0.9% NaCl (Miyoshi et al., 2008). Although the intracellular second messengers, such as cyclic AMP and cyclic di-GMP, and the global general regulators including RpoS and histone-like nucleotide structuring protein are also involved in the gene regulation (Benitez et al., 2001; Wang et al., 2011, 2012), the molecular mechanisms how the bacterium senses the environmental signals and transmits the signals into the cells are not clarified.

Production of proteolytic enzymes is tightly dependent on the growth phase and reaches to the maximum level at the early stationary phase. Many pathogenic bacteria coordinate expression of the virulence genes in response to the bacterial cell density. This regulation system is termed the quorum sensing (QS) and is controlled by the small diffusible signal molecule called autoinducer (AI; Whitehead et al., 2001; Henke and Bassler, 2004). At the low cell density, the QS system cannot modulate the gene expression because the concentration of AI is too low. However, at the high cell density, the AI concentration around the bacterial cell reaches the threshold level, the AI molecule is sensed by the sensor protein, the signals are transmitted into the cell, and finally, the expression of a set of genes is started or stopped.

In *Vibrio* species including *V. cholerae*, *V. mimicus*, and *V. vulnificus* (Miller et al., 2002; Milton, 2006; Sultan et al., 2006), the AI molecule is detected by the specific membrane-bound sensor protein, which causes conversion of the sensor protein from kinase to phosphatase. Subsequently, the sensor protein/phosphatase mediates dephosphorylation of LuxU-LuxO, the response regulator proteins. The dephosphorylated LuxO has no activity to inhibit LuxR or its homolog, the master transcriptional regulator for the genes under the control of the QS system. Therefore, at the high cell density, the transcriptional status of the target genes is changed by the function of LuxR or its homologue. Production of the proteolytic enzymes by pathogenic vibrios is closely related with the extracellular AI level (Kim et al., 2003; Kawase et al., 2004; Raychaudhuri et al., 2006). For instance, the mutant of the AI synthetase showed apparently reduced production of vibriolysin. Therefore, the QS system markedly controls the expression of the proteolytic genes in *Vibrio* species. However, it should be noted that the QS system of *V. cholerae* or *V. mimicus* is operated sufficiently at 37°C (Sultan et al., 2006), whereas, the system of *V. vulnificus* is operated effectively at 26°C but not at 37°C (Miyoshi et al., 2006).

## CONCLUSION

The proteolytic enzymes produced by human pathogenic *Vibrio* species may play a variety of pathological roles: direct roles by digesting many kinds of host proteins or indirect roles by processing other toxic protein factors. Especially, vibriolysin from *V. vulnificus* is thought to be a major virulence factor. However, some contradictions of the pathogenic roles were also reported (Shao and Hor, 2000; Sun et al., 2006). It must be mentioned that the purified enzymes from *V. vulnificus* and *V. proteolyticus*, a non-pathogenic species, are difficult to distinguish in the *in vivo* actions, because both enzymes are members of vibriolysin and have comparative biochemical and toxic activities. However, it has been demonstrated that the high growing ability of *V. vulnificus* in the mammal host is important for the pathogenicity of the bacterium (Watanabe et al., 2004). In addition, production of the toxic or virulence factors including proteolytic enzymes is tightly regulated by environmental factors, the bacterial cell density and so on. Therefore, the overall experiments from various approaches are necessary for evaluation of the extracellular proteolytic enzymes as the virulence factors.

## Conflict of Interest Statement

The author declares that the research was conducted in the absence of any commercial or financial relationships that could be construed as a potential conflict of interest.
